# Classification of Obesity among South African Female Adolescents: Comparative Analysis of Logistic Regression and Random Forest Algorithms

**DOI:** 10.3390/ijerph21010002

**Published:** 2023-12-19

**Authors:** Ronel Sewpaul, Olushina Olawale Awe, Dennis Makafui Dogbey, Machoene Derrick Sekgala, Natisha Dukhi

**Affiliations:** 1 Public Health, Societies and Belonging, Human Sciences Research Council, Merchant House, 2 Dock Rail Road, Cape Town 8001, South Africa; ndukhi@hsrc.ac.za; 2 Institute of Mathematics, Statistics and Scientific Computing (IMECC), University of Campinas, Campinas 13083-859, Brazil; oawe@unicamp.br; 3 Medical Biotechnology and Immunotherapy Research Unit, Institute of Infectious Diseases and Molecular Medicine, Faculty of Health Sciences, University of Cape Town, Cape Town 7700, South Africa; 4 Non-Communicable Diseases, South African Medical Research Council, Cape Town 7505, South Africa; derrick.sekgala@mrc.ac.za

**Keywords:** obesity, adolescent, girls, South Africa, logistic regression, random forest

## Abstract

Background: This study evaluates the performance of logistic regression (LR) and random forest (RF) algorithms to model obesity among female adolescents in South Africa. Methods: Data was analysed on 375 females aged 15–17 from the South African National Health and Nutrition Examination Survey 2011/2012. The primary outcome was obesity, defined as body mass index (BMI) ≥ 30 kg/m^2^. A total of 31 explanatory variables were included, ranging from socio-economic, demographic, family history, dietary and health behaviour. RF and LR models were run using imbalanced data as well as after oversampling, undersampling, and hybrid sampling of the data. Results: Using the imbalanced data, the RF model performed better with higher precision, recall, F1 score, and balanced accuracy. Balanced accuracy was highest with the hybrid data (0.618 for RF and 0.668 for LR). Using the hybrid balanced data, the RF model performed better (F1-score = 0.940 for RF vs. 0.798 for LR). Conclusion: The model with the highest overall performance metrics was the RF model both before balancing the data and after applying hybrid balancing. Future work would benefit from using larger datasets on adolescent female obesity to assess the robustness of the models.

## 1. Introduction

Previously considered to affect well-developed and high-income countries, obesity is a public health threat that is increasing in low- and middle-income countries (LMICs). The global adult obesity rate has almost tripled in the past four decades. Currently, 39% of adults globally are overweight and 13% obese. Overweight among children and adolescents aged 5–19 years increased from 4% in 1975 to over 18% in 2016 [1]. Like other middle-income countries, South Africa is currently facing a triple burden of malnutrition, highlighted by a co-existence of overweight and underweight and micronutrient deficiencies [2]. This has led to the acceleration of obesity rates in children and adolescents in South Africa, as identified in national surveys. The South African National Health and Nutrition Examination Survey (SANHANES), conducted in 2012, found that among children aged 2–5 years, overweight and obesity were recorded as 17.5% and 4.4%, respectively. In females aged 15–17 years of age, overweight and obesity prevalence was indicated as 19.3% and 8.0%, respectively [3]. The South African Health and Demographic Survey 2016 (SADHS) showed that the prevalence of overweight in children aged under five years was 13.0% [4]. In women aged 15–19 years old, overweight and obesity were recorded at 16.1% and 10.9%, respectively. Overweight and obesity were substantially higher among females than males in both surveys, suggesting the need for more detailed investigations into the high prevalence among females in South Africa.

Obesity is characterized by excessive adipose tissue in the body and has multifactorial and complex aetiology [5]. Obesity (and overweight) has been long considered a lifestyle disease as it may be attributed to behavioral or modifiable risk factors such as unhealthy diets and physical inactivity [6]. The trend of weight gain in children and young adults as they transition into adulthood is a major public health concern [7]. The adolescent years have been considered as the “tipping years”, during which the co-morbidities leading to chronic diseases emerge as short-term health consequences of obesity [8]. These include high blood pressure, diabetes, cancers, stroke, and heart disease [9].

Apart from sedentary lifestyles and unhealthy diets in childhood and adolescence, changes in weight and height also contribute to the development of obesity. Body mass index (BMI) is the most frequently used measure of weight in relation to height and has often been used as a measure of overweight and a proxy measure to indicate obesity in individuals [5]. By measuring BMI, the peak is identified at age one, followed by a decline up to age six, and then a rise known as “adipose rebound” [10]. During these growth periods, both hormonal and metabolic changes largely influence adiposity at various ages [11]. Obesity risk factors during the childhood to adolescence period include family history of obesity, dietary factors such as sugar, fat, and protein consumption, dietary knowledge and preferences, physical activity, socioeconomic environments (household wealth, income, access to nutritious food, and healthcare access), lifestyle behaviors such as substance use, and psychological factors that present during adolescence [2,11,12].

The burden of obesity in health systems is undeniably enormous, necessitating strategies that are aimed at its prediction from childhood through to adulthood [13]. The progression of childhood obesity to adulthood obesity is well established by evidence from large cohorts. Results from a UK study where participants were followed prospectively concluded that more than half (55%) of obese children will progress to be obese during adolescence, and close to 80% of obese adolescents will become obese in their adulthood. Hence, predictor models addressing the risks of obesity progression from childhood to adulthood are needed to inform the development and adaptation of preventive policies [14]. These prediction models may be either statistical or machine learning (ML) models, the latter of which have gained traction in recent years.

LR is the most commonly applied tool in prediction models for public health outcomes, including child obesity and overweight [15]. Conversely, ML models have been gaining increasing acceptance in recent years due to their higher precision rates and their abilities to model complex non-linear relationships between variables and manage high-dimensional data [12,16].

The use of ML to model body mass index (BMI), including overweight and obesity, has been predominantly conducted in adult samples in developed countries [16,17], with relatively few studies on childhood and adolescent obesity. A review of ML studies for child and adolescent obesity is presented by Siddiqui et al. (2021) [18]. Predicting adolescent obesity development requires a nuanced approach due to its multifaceted nature, in addition to the hormonal and metabolic changes during childhood and adolescence.

One of the most commonly used ML methods in many scientific fields is the random forest (RF) algorithm [19]. The RF model is based on groups of decision trees derived from random samples with replacement of the training set and with random subgroups of the explanatory variables used at each split in the decision trees. Averaging the prediction of all the trees in the RF model is used when predicting new data. RF models have been applied in diverse real-world scenarios. These include using credit scoring to predict defaulting and to detect fraudulent transactions in finance and banking, using patient data and medical records in disease diagnosis and drug discovery, customer segmentation in marketing research, demand forecasting in retail, and forecasting equipment failures and demand fluctuations in manufacturing and supply chain management.

RF models have been applied in several studies that apply and compare various ML methods to model overweight in children [20,21,22,23] and adolescents [13,24]. These studies are often classified as predictor-focused or prediction-focused models [18]. The predictor-focused models aim to investigate the importance of factors associated with obesity, while the prediction-focused models are primarily concerned with the accurate prediction of obesity. The latter is the focus of this study.

Logistic and linear regression are sometimes viewed as more interpretable than ML models such as RF and general Neural Networks [18]. A large-scale benchmarking experiment comparing the prediction performance of the RF algorithm with LR showed that the RF model performed better than LR with respect to AUC, accuracy, and the Brier score [25]. LR is often used with low-dimensional data, that is, when the number of covariates is modest relative to the sample size. However, comparisons of machine learning prediction models with traditional statistical methods, like LR for modelling obesity/overweight, are rare. A literature search of studies comparing LR and ML methods that included random forests to predict obesity and overweight revealed only a few studies, the findings of which are summarized below.

Evidence from a Bangladeshi study among individuals of all ages found that the LR algorithm achieved the highest accuracy (97.1%) in obesity prediction compared to the other ML models, including RF models [26].

Zhang et al. (2009) [27] compared the results of LR with those of six popular data mining techniques to predict overweight and obesity in children and found that LR and decision tree methods achieved relatively poor predictive rates.

A comparison of RF and LR methods to determine the most relevant risk factors for overweight in Finnish adults found that the RF model did not have a higher power in variable selection when compared to LR. The authors noted that the RF model would be more beneficial when using a larger number of explanatory or predictor variables [28].

Molina Estren et al. (2021) [29] used Logistic Model Tree methods (a combination of decision trees and LR) and the RF model to classify obesity in young adults and found that Logistic Model Tree had better performance in precision (96.6%) compared to random forest methods (95.6%).

Siddiqui et al. (2021) [18] suggest that the gender-specific and ethnicity-specific models would result in higher prediction performance because the development of obesity is different for boys and girls and for different populations or ethnic groups. This study thereby bridges this gap in knowledge by comparing the RF algorithm with LR to predict obesity in a cross-sectional sample of adolescent females in South Africa.

## 2. Materials and Methods

### 2.1. Dataset Description

Data was extracted from the South African National Health and Nutrition Examination Survey (SANHANES), a cross-sectional national household survey conducted in 2011/12. The survey investigated the health and nutritional status of South Africans. Data was collected via interviews, physical examination, and blood samples for biomarker analyses. A multistage disproportionate, stratified cluster sampling approach was used, where 1000 census enumerator areas (EAs), selected from the 2001 population census (86,000 EAs), were mapped in 2007 using aerial photography for the creation of the 2007 Human Sciences Research Council (HSRC) master sample. EA selection was stratified by province and locality. Overall, 500 EAs were selected, and 20 visiting points/households per EA were selected, resulting in a sample of 10,000 households. Of the sampled households, 8166 were occupied. Within the occupied households, 27,580 individuals were eligible and agreed to be interviewed, of whom 25,532 completed the interview and 12,025 underwent physical examinations. Further details on the survey methodology, indicators and data collection are available in the SANHANES report [3].

**Figure 1:**
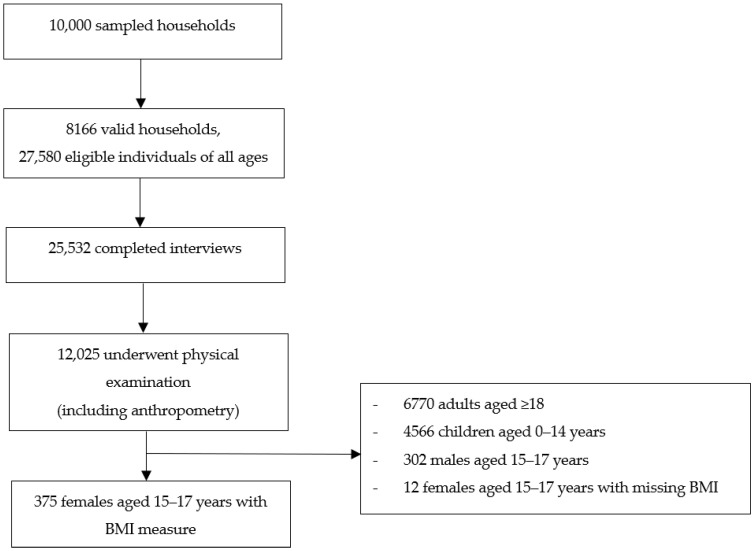
Participant flow chart.

The physical examination included the obtaining of anthropometric measures. In this study, data were analysed on adolescent girls aged 15–17 years for whom anthropometric measures were obtained in the physical examination. Figure 1 illustrates the participant flow chart. Covariates relevant to the risk factors for obesity (based on a review of the literature) [1,2,11,12] were extracted. The extracted dataset used in this study consisted of 375 records and 32 attributes, of which 31 attributes are explanatory features or covariates, and one attribute is the primary outcome variable.

### 2.2. Measures

#### 2.2.1. Primary Outcome

The primary outcome variable was obesity. Heights and weights were measured using standardised techniques described by Lee and Nieman (2013) [30]. Body mass index (BMI) was calculated as weight (in kg) divided by the square of height (in meters). The BMI-for-age (designated as a percentile) cut-offs for 15–17-year-olds from the Centers for Disease Control (CDC) [31] were used to categorise participants as underweight (BMI < 18 kg/m^2^), normal (18–24.99 kg/m^2^), overweight (25–29.99 kg/m^2^), and obese (≥30 kg/m^2^). The BMI categories were recoded into a binary variable where underweight, normal weight and overweight were classified as 0 = ‘not obese’ and 1 = ‘obese’.

#### 2.2.2. Explanatory Covariate Variables

The explanatory covariates were grouped into four domains: demographic, socioeconomic, dietary variables, behavioural risk factors, family history of non-communicable diseases (NCDs), and blood pressure variables.

The four demographic variables were age (denoted ‘AGEfinal’ in the dataset), province (‘province’), race group (‘race’), and locality type (‘geotype’). Race was reported using the standard population groups from Statistics South Africa [32]. Locality type was derived from the EA in which a participant’s household was situated. It comprised urban informal, urban formal, rural informal (traditional tribal areas), and rural formal (farm areas).

Socioeconomic covariates comprised seven variables, namely household income (‘hhinc2′), household wealth index (‘aindex_cat’), dwelling type (‘dwell_typ’), health insurance (‘medaid’), household engagement in meat or poultry agriculture (‘agric_animal’), household food insecurity (‘hunger_cat’), and access to healthcare in the past two years (‘healthcare_access2yr’). Household income referred to the per capita household income divided into tertiles for low, intermediate, and high incomes, and a fourth option for when income was not reported. The household wealth index was based on the Filmer-Pritchett asset wealth index. It was calculated using Principal Component Analysis (PCA) with sixteen variables on housing type, water services, sanitation services, and ownership of 13 household assets. The index was grouped into chronic poor (0–50th percentile), vulnerable poor (51–75th percentile), middle-income (76–95th percentile), and rich (96–100th percentile) [33]. Household dwelling type referred to formal or informal housing types. Medical aid was based on the household members having health insurance. Household engagement in meat or poultry agriculture referred to the household using these forms of agriculture. Household food insecurity was measured by the Community Childhood Hunger Identification Project (CCHIP) [34], which uses eight questions on adults and/or children in the household being affected by food shortages, perceived food deficiency, or changes in food intake due to limited economic resources in the household. A score of 0 indicates a household that is food secure, scores of 1–4 indicate a household at risk of hunger, and scores of 5–8 indicate that the household is experiencing hunger. Access to healthcare in the past two years was based on individual participant responses to whether they had consulted a healthcare provider during the preceding two years.

There were nine dietary variables, namely dietary diversity score (‘DDScat’), nutrition knowledge (‘NutriKnowA_cat’), sugar consumption (‘sugarscore_cat’), fruit and vegetable consumption (‘fruitscore_cat’), fat consumption (‘CategoricalFatScore’), consumption of red meat with the fat on (‘redmeat_wfat’), daily milk consumption (milkserv_daily), preference of fat spreads (‘butterspread’), and frequency of snack consumption per day (snack_freq). The dietary diversity score (DDS) was derived from individual participants’ 24-h recall of the foods and drinks they had consumed the previous day. The foods were divided into nine food groups, and a sum score was calculated from the number of food groups they had consumed. A DDS of less than four is low and considered to be linked to dietary inadequacies [35]. The nutrition knowledge score was based on a sum score of nine questions on knowledge about fibre, fat, sugar, and fruit in the diet. Scores of 0–3 correct answers were considered low, 4–6 as moderate, and 7–9 as high nutrition knowledge. Sugar consumption was measured by four items on the frequency of consumption of sugary foods like sweetened beverages and confectionery in the past week. A sum score was computed where scores of 5–8 were considered high sugar consumption, 3–4 as moderate, and 0–2 as low. Fat consumption was measured by the sum score of ten items on the frequency of past-week consumption of high-fat foods, and the sum scores of 11–20, 6–10, and 0–5 were categorised as high, moderate and low-fat consumption, respectively. Fruit and vegetable consumption included four questions on the frequency of consumption of vegetables and fresh fruit in the past week. Based on the data distribution of the sum score, scores of 5–8, 3–4, and 0–2 were considered high, moderate, and low consumption, respectively. Daily milk consumption was based on the amount of milk consumed on an average day. Preference for fat spreads was assessed by the participants’ reported preference of how much butter, fat, or margarine they usually spread on bread or crackers. Red meat consumption was assessed by whether participants consumed red meat with or without the fat removed. Snack frequency was based on the number of meals and snacks per day, where three or more meals with snacks in between was considered high snack frequency.

The other behavioural risk factors were the following six variables: physical activity (‘physical_activity’), weight loss attempts (‘weightloss_attempt_pastyear’), weight gain attempts (‘weightgain_attempt_pastyear’), current smoking (‘Cursmoker’), high alcohol consumption (‘auditc3_mf1′), and psychological distress (‘psych_dist2′). Physical activity was based on the WHO Global Physical Activity Questionnaire (GPAQ), which categorises less than 2000 Metabolic Equivalents (MET) minutes per week as high physical activity [36]. High-risk alcohol use was measured using the AUDIT-C, a 3-item alcohol screening tool [37]. Current tobacco smoking was self-reported. Weight loss and weight gain attempts were based on whether participants had attempted to lose or gain weight in the past year. The Kessler-10 [38], a 10-item questionnaire, measured psychological distress. A sum score of ≥20 is considered mild to severe psychological distress, and a score of <19 is considered minimal distress.

Three variables measured family history of NCDs, namely family history of high blood pressure (‘famhist_hbp’), family history of diabetes (‘famhist_diabetes’), and family history of heart disease (‘famhist_heartdis’). They were each based on a question of whether they had a close blood relative who had each of these conditions.

The blood pressure variables were systolic blood pressure (‘SBPfinal’) and diastolic blood pressure (‘DBPfinal’). Three systolic and diastolic blood pressure measurements were ascertained after 5–10 min of rest using an Omron Automatic Digital BP monitor (model M2, Omron Healthcare, Bannockburn, IL, USA). The average of the second and third measures were used as the final reading.

### 2.3. Analysis

Analyses were performed using the R software and studio. Multiple Imputation by Chained Equations (MICE) was used to impute the missing data values in the original dataset. The percentage of missing values across all the covariates ranged from 4% to 28%. The MICE procedure, a robust method of dealing with missing data, imputes missing data values using iterative predictive models. Each variable in the dataset is imputed using the other variables in successive iterations. The LR and RF algorithms were run on the imputed dataset. The dataset was divided into training/test subsets in a ratio of 70/30.

The dataset is considered imbalanced because the number of individuals without the primary outcome of interest, that is, who were not obese, is much larger than the number of individuals who were obese. Therefore, we performed resampling techniques to address the class imbalance in the data. These were oversampling, undersampling, and a hybrid (both under- and oversampling) on the data. Experimentation of the LR and RF algorithms was performed on the imbalanced data as well as on the dataset after oversampling, undersampling and hybrid sampling [39,40].

The confusion matrix was used to evaluate the performance of the classification models with respect to correctness and accuracy. The elements of the CM used include True Positive (TP), True Negative (TN), False Positive (FP), and False Negative (FN). Precision, recall, F1-score, and balanced accuracy were also calculated. Precision refers to the proportion of data predicted as true that is actually true and is given by the formula Precision = TP/(TP + FP). Recall (or Sensitivity) calculates the percentage of data that is actually positive despite the fact that it was forecasted to be positive. The formula for calculating recall is given by TP/(TP + FN). The F1 score is considered the harmonic mean of precision and recall and is, therefore, a composite measure of both. Youden’s Index, calculated as Sensitivity + Specificity − 1, identifies the optimal cutoff point within the model by maximising the difference between true positive and false positive rates. Balanced accuracy is the average of sensitivity and specificity. Specificity is the proportion of people without obesity who are identified as such and is given by TN/(TN + FP). Balanced accuracy is good for capturing data imbalance. McNemar’s test *p*-value is also calculated, where the null hypothesis is the homogeneity of the proportion of misclassified cases for the two classes.

## 3. Results

### 3.1. Description of the Sample

Of the 375 females aged 15–17 years, 8.3% (n = 31) were obese. Less than half lived in urban formal areas (43.2%), 80.3% lived in formal dwellings, 38.1% lived in households that were food secure, and 37.1% had accessed healthcare in the past two years. A quarter of the females had high sugar consumption, a third had low fruit and vegetable consumption, 55.7% had high dietary diversity scores, 13.1% had tried to lose weight during the past year, and 78.4% had low rates of physical activity (Table 1).

### 3.2. Performance of the Models

Table 2 shows the performance metrics of the two algorithms using the imbalanced data as well as using under-sampling, oversampling and hybrid sampling of the datasets. The confusion matrix was used to derive each model’s performance metrics. Using the imbalanced data, the RF algorithm performed better with higher precision, recall, F1 score, and balanced accuracy. McNemar’s test *p*-value was >0.05 for the RF model only, which showed that the RF model classifies similar proportions of errors on the test set.

**Figure 2:**
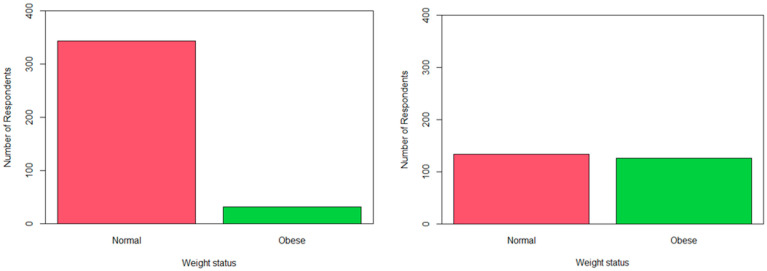
Distribution of the obesity variable using the imbalanced data (**left panel**) and the hybrid balanced data (**right panel**).

The oversampled data resulted in lower precision, recall, F1 score, and Balanced accuracy than the Imbalanced data for both the RF and LR models. The undersampled data resulted in much lower recall and F1 scores but higher balanced accuracy for the RF model. The balanced accuracy was highest with the hybrid data (both over- and undersampling of the data) at 0.618 for the RF model and 0.668 for LR. Figure 2 shows the distribution of the obesity variable in the imbalanced data compared to the hybrid balanced data. Using the hybrid balanced data, the RF model performed better in terms of higher recall, *p*-value and F1-score.

**Figure 3:**
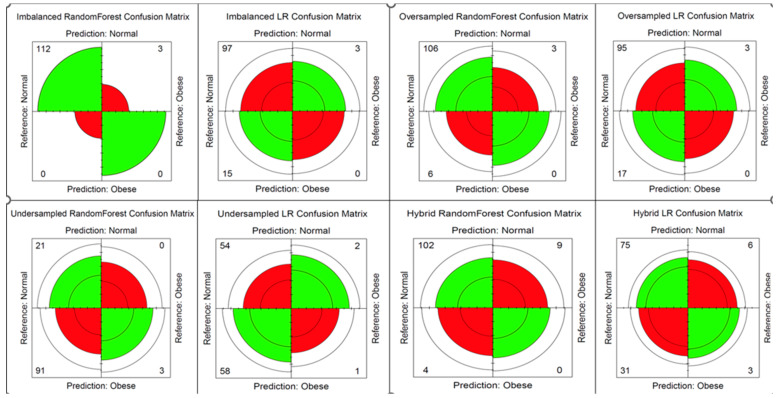
Confusion matrices for the RF and LR models before and after hybrid, under-sampling and oversampling of the data.

The RF models took longer times to run than the LR. The RF model using the hybrid sampling took 81.98 s, which was much shorter than the RF model using the imbalanced dataset. The confusion matrices are displayed in Figure 3, where the bottom left and top right panes in each matrix are the incorrectly classified normal weight and obese participants, respectively.

**Figure 4:**
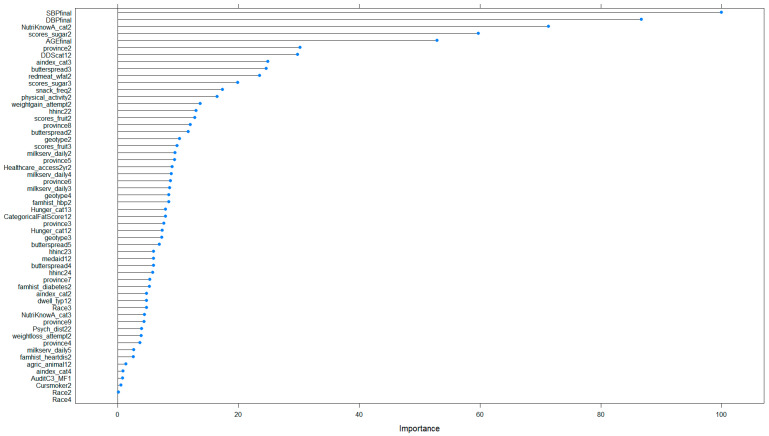
Variable importance of the variables used in the random forest model with hybrid balancing.

The RF model, after hybrid sampling, identified blood pressure, nutrition knowledge, sugar consumption and age as having the highest importance in predicting obesity in this sample of adolescent females (Figure 4).

As a secondary/sensitivity analysis, the models on RF and LR before and after over-, under- and hybrid sampling were applied to another subset of the SANHANES data on adolescent males and females (n = 671) to predict overweight. Overweight was defined as BMI ≥ 25 kg/m^2^. In these models, the RF model performed well in terms of higher F1 scores than LR (Supplementary Table S1).

## 4. Discussion

The study is the first to evaluate and compare RF and LR models for predicting obesity in South African female adolescents. The study showed that RF performed better than LR in predicting obesity in adolescent females in terms of higher precision, recall, F1 score and balanced accuracy when using the imbalanced data. After hybrid balancing, the RF model also performed better than LR in terms of higher recall, *p*-value, and F1 score. Therefore, in this study, machine learning-based algorithms showed better prediction accuracy than the statistical-based algorithm. Future work would benefit from using a larger dataset to assess the robustness of the models.

The hybrid balancing resulted in the highest balanced accuracy when compared to the oversampling and under-sampling techniques and when compared to the imbalanced data. Under sampling resulted in much lower performance metrics than oversampling, a finding that has been previously shown [40]. Hybrid techniques have performed better in addressing class imbalance than oversampling and under sampling alone [41], especially in the case of extremely imbalanced data when the numbers of one class far outweigh the other class [42], as was the case in the obesity data used in this study.

The variables that had the most importance in predicting obesity among adolescent females using the RF model after hybrid sampling were blood pressure, nutrition knowledge, sugar consumption, and age. The literature shows a clear link between obesity and hypertension in adolescent populations [43]. Weight loss interventions aimed at children and adolescents are key to reducing blood pressure [44]. Nutrition knowledge influences food choices and dietary behaviors and has also been shown to be associated with obesity in children and adolescents [45].

In practical applications, RF models can be used to detect and diagnose obesity using electronic health records that capture various patient characteristics such as demographics, lifestyle factors like diet, exercise, and sleep patterns, as well as health metrics or biomarkers like blood pressure and medical history. The RF model, therefore, finds use in clinical settings for early intervention, in public health programs for targeted interventions, and in mobile wellness apps that deliver personalized insights. RF models can be implemented through various techniques, such as API integration, containerization, and continuous monitoring, to ensure that they are accurate and adaptable to changing data. Early detection of obesity or the risk thereof, particularly in adolescents, allows healthcare providers to intervene at an early age using health behavior modification programmes for weight loss.

The strengths of the study include its wide range of explanatory variables that cover multiple domains, including socio-economic, psychosocial, demographic, family history, physical activity, and dietary variables. However, a limitation is that variables from other important domains that are known to influence adolescent obesity development, such as parent characteristics and environmental and health-related variables during early childhood and in-utero, were not captured in the SANHANES survey. Hence, the prediction of obesity in this study is limited to only the variables in the dataset. Future work in this area would benefit from expanding the domains of explanatory variables, as this is likely to improve precision in predictive models.

Secondly, the study is based on cross-sectional data, which limits findings of causality. The study is also based on a relatively small sample of adolescents. Interpreting both RF and LR models for obesity data also comes with inherent limitations. LR assumes a linear relationship between covariates and the log odds of the obesity outcome, which may fail to model complex nonlinear patterns. RF models also present challenges in interpretation, where it is difficult to precisely understand the relationships between covariates and the likelihood of obesity. In addition, the RF model faces limitations in extrapolating beyond the range of the training data, impacting its reliability with test data containing values outside the training range.

A further strength of the study is the modelling of adolescent girls separately instead of pooling both boys and girls together and its use of anthropometric measurements obtained through standardized procedures. These are considered strengths in light of a recent review of ML models for obesity in children and adolescents that found that most studies do not develop gender-specific models and include self-reported heights and weights to derive BMI [18]. Furthermore, the study was conducted using data from South Africa, a developing middle-income country, whereas the majority of studies have been conducted in high-income, developed countries.

## 5. Conclusions

This study explored the efficacy of LR and RF algorithms in classifying obesity among South African female adolescents. It found that the RF model predicted obesity with better performance than LR both before and after addressing class imbalance in the data, underscoring the efficacy of RF in capturing complex relationships within the dataset used. Blood pressure, nutrition knowledge, sugar consumption, and age were important variables in predicting obesity, underscoring the significance of blood pressure screening in young people and for health promotion interventions aimed at improving dietary knowledge and subsequent dietary behaviours among youth. The superior performance of the RF model, coupled with the identification of influential predictors, emphasizes the potential for precise modeling of obesity in adolescent populations. This study lays a foundation to encourage future research to refine obesity classification models and design targeted interventions that address the identified determinants, fostering healthier lifestyles among South African female adolescents. Obesity classification models hold promise in guiding public health initiatives and policies, steering towards a proactive approach to combat obesity and promote well-being among adolescent populations globally.

## Figures and Tables

**Table 1:** Description of the sample.

	%	Frequency
Obesity		
Not obese	91.7	344
Obese	8.3	31
Demographic characteristics		
Province		
Western Cape	13.6	51
Eastern Cape	14.7	55
Northern Cape	4.8	18
Free State	8.5	32
KwaZulu Natal	16.8	63
North West	10.7	40
Gauteng	8.3	31
Mpumalanga	12.8	48
Limpopo	9.9	37
Locality type		
Urban formal	43.2	162
Urban informal	13.3	50
Rural informal (tribal)	30.9	116
Rural formal (Farms)	12.5	47
Age		
15	33.9	127
16	28.3	106
17	37.9	142
Race		
African	73.6	276
White	1.3	5
Coloured	21.9	82
Indian	3.2	12
Socioeconomic characteristics		
Household income		
Lower	32.3	121
Intermediate	32.8	123
Upper	22.1	83
Unknown	12.8	48
Household wealth index		
Poor	57.6	216
Vulnerable	29.1	109
Middle	12	45
Rich	1.3	5
Dwelling type		
Formal	80.3	301
Informal	19.7	74
Has medical aid		
Yes	12.5	47
No	87.5	328
Household engages in animal agriculture		
Yes	6.1	23
No	93.9	352
Household food security		
Food secure	38.1	143
At risk of hunger	22.9	86
Experience hunger	38.9	146
Accessed healthcare in the past 2 years		
Yes	37.1	139
No	62.9	236
Dietary variables		
Sugar consumption		
Low (0–2)	31.2	117
Moderate (3–4)	44.8	168
High (5–8)	24	90
Fruit and vegetable consumption		
Low (0–2)	33.3	125
Moderate (3–4)	45.1	169
High (5–8)	21.6	81
Nutrition Knowledge score		
Low (0–3)	14.7	55
Moderate (4–6)	69.3	260
High (7–9)	16	60
Dietary diversity score		
High (4–9)	55.7	209
Low (0–3)	44.3	166
Fat consumption		
High (8–20)	50.4	189
Low (0–7)	49.6	186
Eats red meat with/without fat		
Eats red meat with fat	65.9	247
No red meat or eats red meat without fat	34.1	128
Daily milk consumption		
None	31.7	119
Less than half cup	6.4	24
Half–1 cup	21.9	82
1–2 cups	34.1	128
>2 cups	5.9	22
Snack frequency		
High snack frequency per day	36	135
Low snack frequency per day	64	240
Preference of butter spread		
None	8.3	31
Very thin/scraped on	33.3	125
Thin (just covered)	26.4	99
Medium (nicely covered)	24.5	92
Thick (see teeth marks)	7.5	28
Other behavioral risk factors		
Weight loss attempts		
Yes	13.1	49
No	86.9	326
Weight gain attempts		
Yes	14.7	55
No	85.3	320
Risky alcohol use		
Low	93.6	351
High	6.4	24
Current tobacco smoking		
Current smoker	3.2	12
Non-current smoker	96.8	363
Psychological distress		
Mild-severe distress	10.4	39
No-minimal distress	89.6	336
Physical activity		
High activity	21.6	81
Low activity	78.4	294
Family history of NCDs		
Family history of high blood pressure		
Yes	23.7	89
No	76.3	286
Family history of diabetes		
Yes	20.5	77
No	79.5	298
Family history of heart diseases		
Yes	5.9	22
No	94.1	353
Blood pressure variables		
Systolic blood pressure (mmHg) (Mean, S.D.)	114.9	11.7
Diastolic blood pressure (mmHg) (Mean, S.D.)	66.1	8.6

S.D.—standard deviation; NCDs—non-communicable diseases.

**Table 2:** Performance metrics of the random forest and logistic regression models before and after hybrid, under-sampling and oversampling of the data.

Metrics	Imbalanced		Oversampled		Under-Sampled		Hybrid	
	Random Forest	Logistic Regression	Random Forest	Logistic Regression	Random Forest	Logistic Regression	Random Forest	Logistic Regression
Precision	0.974	0.970	0.973	0.969	1.00	0.964	0.981	0.987
Recall	1.00	0.866	0.946	0.848	0.188	0.482	0.902	0.670
F1 Score	0.987	0.915	0.959	0.905	0.3158	0.643	0.940	0.798
Balanced accuracy	0.500	0.433	0.473	0.424	0.594	0.408	0.618	0.668
Mcnemar’s Test *p*-Value	0.248	0.010	0.505	0.0037	<2 × 10^−16^	1.24 × 10^−12^	0.027	1.37 × 10^−8^
Youden’s Index	0	0.8661	−0.0536	−0.1518	0.1875	−0.18453	0.23512	0.33631
Time to run model (seconds)	102.19	77.16	145.3	35.51	25.89	27.86	81.98	21.17

## Data Availability

Data used in this study are available at: https://repository.hsrc.ac.za/handle/20.500.11910/13690 (accessed on 10 January 2022).

## Supplementary Materials

The following supporting information can be downloaded at: https://www.mdpi.com/article/10.3390/ijerph21010002/s1, Table S1: Performance metrics of the random forest and logistic regression models using data on SAN-HANES adolescent males and females to model overweight.
