# Supplementary material for: Classification of Obesity among South African Female Adolescents: Comparative Analysis of Logistic Regression and Random Forest Algorithms
Source: Int J Environ Res Public Health. 2023 Dec 19;21(1):2. doi: 10.3390/ijerph21010002 (PMC10815679; doi:10.3390/ijerph21010002)
Supplement: Supplementary file 1 [file ijerph-21-00002-s001.zip › ijerph-2670034-supplementary.pdf]

Supplementary file

Table S1. Performance metrics of the random forest and logistic regression models using data on SANHANES adolescent males and females to model overweight.

Metrics	Imbalanced		Oversampled		Under-sampled		Hybrid	
	Random Forest	Logistic Regression	Random Forest	Logistic Regression	Random Forest	Logistic Regression	Random Forest	Logistic Regression
Precision	0.794	0.810	0.804	0.792	0.875	0.871	0.796	0.849
Recall	0.969	0.914	0.933	0.632	0.558	0.620	0.791	0.656
F1 Score	0.873	0.859	0.864	0.703	0.682	0.724	0.794	0.741
Balanced accuracy	0.508	0.550	0.536	0.502	0.628	0.635	0.512	0.607
Mcnemar's Test P-Value	0.000	0.004	0.000	0.001	0.000	0.000	1.000	0.000
Time (seconds)	109.810	39.030	151.920	32.050	71.650	30.670	86.740	26.330
